# Cumulative blood pressure exposure and cognition: the potential mediating role of brain volume

**DOI:** 10.1038/s41440-025-02534-z

**Published:** 2026-01-14

**Authors:** Xiaoshuai Li, Zejun Zhu, Ying Hui, Huijing Shi, Jiacheng Fan, Wei Hong, Xiaohui Hu, Xianyu Zhu, Haitao Li, Lingmei Yue, Shun Zhang, Xiaoliang Liang, Shuohua Chen, Han Lv, Pengfei Zhao, Jing Li, Yuntao Wu, Zhenjian Yu, Shouling Wu, Zhenchang Wang

**Affiliations:** 1 Department of Radiology, Beijing Friendship Hospital, Capital Medical University, Beijing, China; 2 Kailuan Mental Health Center, Tangshan, China; 3 Department of MR, Kailuan General Hospital, Tangshan, China; 4 Department of Rheumatology and Immunology, Kailuan General Hospital, Tangshan, China; 5 Precision and Intelligence Medical Imaging Lab, Beijing Friendship Hospital, Capital Medical University, Beijing, China; 6 Department of Neurology, Beijing Friendship Hospital, Capital Medical University, Beijing, China; 7 Beijing Tsinghua Changgung Hospital, Beijing, China; 8 Department of Cardiology, Kailuan General Hospital, Tangshan, China

**Keywords:** Cumulative blood pressure, Brain structure, Perfusion, Cognitive function

## Abstract

Elevated blood pressure (BP) has been linked to brain structure changes and cognitive decline. However, few studies have accounted for long-term cumulative BP exposure. We investigated the association between cumulative BP exposure, brain volume, cerebral blood flow (CBF), and cognitive decline. Furthermore, we explored whether alterations in brain volume and CBF mediated the association between cumulative BP and cognitive decline. We included 1012 adult participants from the Kailuan study. Cumulative BP exposure was calculated from 2006 to 2020. Brain MRI scans and the Montreal Cognitive Assessment (MoCA) were performed in 2020. Generalized linear regression models were used to investigate the associations between cumulative BP, brain volume, CBF, and cognitive function. Mediation analysis was performed to examine whether alterations in brain volume and CBF mediated the association between cumulative BP and cognitive decline. Compared with the lowest tertiles, the highest tertiles of cumulative SBP were associated with lower volumes in total brain (−9.11 [−16.25, −1.97]), total GM (−5.53 [−10.02, −1.04]), frontal lobe (−2.46 [−4.15, −0.78]), temporal lobe (−1.37 [−2.51, −0.23]) and hippocampus (−0.15 [−0.26, −0.03]), and the highest tertiles of cumulative DBP were associated with lower volume in frontal lobe (−2.33 [−3.98, −0.68]) and temporal lobe (−1.15 [−2.27, −0.04]). Higher cumulative SBP and DBP were associated with lower total and regional CBF and MoCA scores (all *P* < 0.05). The associations between cumulative DBP and cognitive decline were mediated by the volumes in total GM, frontal lobe and temporal lobe. Early intervention in cumulative BP may help preserve brain structure and function.

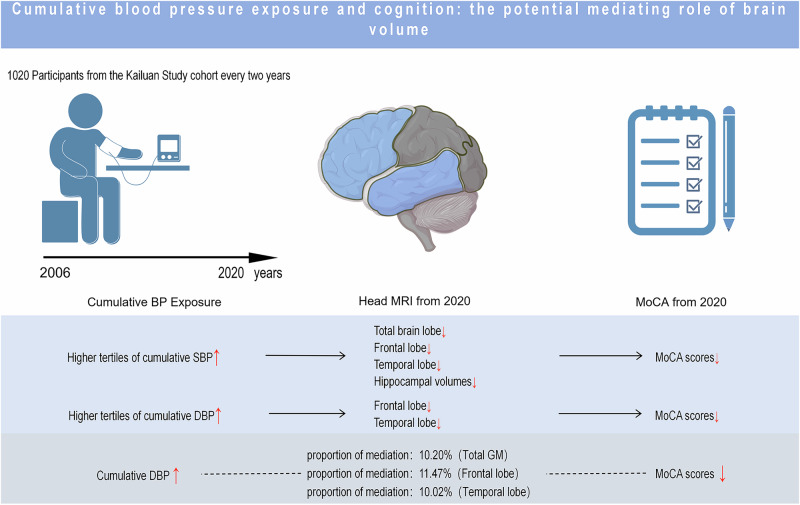

## Introduction

With improvements in social progress, living standards and medical conditions, the global aging population has increased significantly, which is accompanied by an increased prevalence of cognitive decline and dementia [1]. The pathological basis of cognitive decline is changes in brain structure [2]. The maintenance of normal brain structure and function requires a good blood supply to the brain and the clearance of metabolites [3]. The main factor that ensures a normal cerebral blood flow (CBF) supply is maintaining blood pressure (BP) within a certain range; a BP that is too high or too low can affect the cerebral blood supply and subsequently damage brain structure and impair brain function [4]. Previous studies have shown that hypertension is a risk factor for structural brain damage and cognitive decline, but most studies have analyzed the associations between a single BP value and structural brain damage or cognitive decline [5].

Cumulative exposure accounts for not only the cumulative exposure to risk factors but also the duration of exposure. Compared with a single exposure, cumulative exposure is more reliably associated with adverse outcomes. Previous studies have shown that cumulative BP, fasting blood glucose (FBG), blood lipid and body mass index (BMI) exposures are more strongly associated with atherosclerotic cardiovascular disease than are single measurements of BP, FBG, blood lipids and BMI [6–9]. Mahinrad S. et al. reported that higher cumulative BP exposure is a risk factor for cognitive decline [10]. In theory, cumulative BP exposure could impair cognitive function by affecting brain structure [10]. To confirm the above hypothesis, we used multimodal brain imaging data embedded in the Kailuan study to systematically explore the associations among cumulative BP exposure, brain volume, CBF, and cognitive function and to further analyze whether brain alterations mediate the association between cumulative BP exposure and cognitive decline.

## Methods

### Participants

**Fig. 1:**
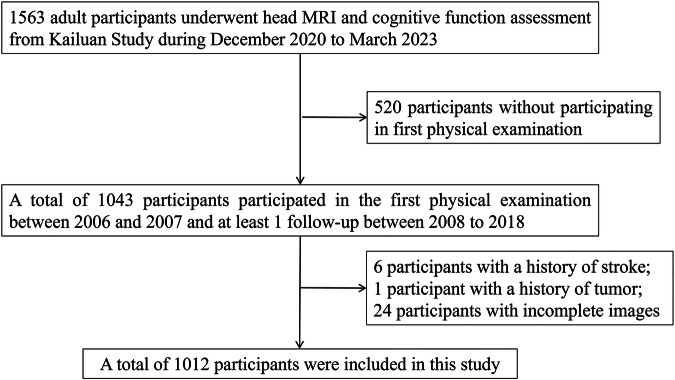
Flowchart of enrolment for participants in this study

The study was approved by the ethics committees of Kailuan General Hospital. All participants provided informed consent in accordance with the protocol. A prospective cohort study was conducted by the Kailuan Study in Tangshan, China [11]. From July 2006 to October 2007, a total of 101,510 individuals older than 18 years from the Kailuan community were included in the first survey and followed up every 2 years. The questionnaire assessment, clinical assessment and laboratory examination were conducted by professionals. The seventh follow-up began in December 2020, and some participants underwent head magnetic resonance imaging (MRI) and cognitive function assessment on the basis of the original protocol. The criteria for the inclusion of participants in this study were as follows: (1) participated in the 7th follow-up and underwent cognitive function assessment and head MRI examination beginning in December 2020; (2) underwent the first physical examination between 2006 and 2007; and (3) participated in at least 1 follow-up visit between 2008 and 2018. Participants with a history of tumor, stroke or incomplete imaging data were excluded. The flow chart is shown in Fig. 1. Overall, 1012 participants were included in the study.

### Cumulative BP exposure

Systolic BP (SBP) and diastolic BP (DBP) were measured by trained professionals via a digital BP monitor (Omron HBP-1100U). After the participants sat in a quiet room for 5 min, the BP of the right brachial artery was measured. The average value of 3 measurements was taken for analysis. The cumulative BP exposure (unit: mm Hg × years) for each participant was calculated as the sum of the mean BP of two consecutive follow-up visits multiplied by the time interval between follow-up visits (unit: year) [12]: cumulative BP = [BP2006 + BP2008] / 2 × [Visit2008 − Visit2006] + [BP2008 + BP2010] / 2 × [Visit2010 − Visit2008] + …… + [BP2018 + BP2020] / 2 × [Visit2020 − Visit2018]). The cumulative SBP and DBP were calculated.

### MRI data acquisition

All participants were scanned via a 3.0 T MRI scanner (GE, 750 W, Milwaukee, WI, USA). The imaging protocol included 3D T1-brain volume images (echo time (TE) = 2.6 ms; repetition time (TR) = 6.7 ms; flip angle (FA) = 15°; slice thickness = 1 mm; field of view (FOV) = 256 mm × 256 mm; gap = 1 mm; 170 slices) and 3D pseudocontinuous arterial spin labeling (3D-pc ASL) images (TR = 5313 ms; TE = 10.7 ms; slice thickness=4 mm; flip angle=111°; in-plane resolution, 3.37 mm × 3.37 mm; FOV = 256 mm × 256 mm; and postlabel delay = 2525 ms).

### MRI data processing

3D-PC ASL images were processed via SPM12 as described previously [13]. The CBF maps were automatically generated via 3D ASL Functool software (AW4.6 Workstation, GE Healthcare). The CBF images were subsequently registered into the MNI space. Finally, the average CBF values of the total brain and areas of interest (frontal lobe, parietal lobe, temporal lobe and hippocampus) closely related to cognition were extracted (Supplementary Fig. 1) [14, 15].

SPM12 software was used to preprocess the 3D T1-brain volume image data. The detailed processing steps, including reorientation, segmentation, and normalization, have been described in previous studies [16]. Finally, total GM volume and white matter volume were computed for all participants. The total brain volume was defined as the sum of the total GM volume and white matter volume [17]. Additionally, regional brain volumes (frontal lobe, parietal lobe, temporal lobe, and hippocampus) closely related to cognition were extracted (Supplementary Fig. 1).

### Covariate measurement

The demographic characteristics of the participants, including age, sex, medical history and family medication information, were collected by trained professionals via standard questionnaires. The participants self-reported their lifestyle habits, including smoking status, alcohol consumption status and physical exercise. The physical examination involved an evaluation of weight, height, and BMI. Laboratory tests were conducted by using venous blood samples collected at 7–9 a.m. on the second day following a 12-hour fast. Biochemical markers, including FBG, triglyceride, total cholesterol, low-density lipoprotein cholesterol (LDL-C), and high-density lipoprotein cholesterol levels, were measured via an automated analyzer (Hitachi 747; Hitachi Ltd., Tokyo, Japan).

### Cognitive function assessment

Cognitive function was assessed by psychiatrists via the Montreal Cognitive Assessment (MoCA). This scale covers major cognitive functions, including visuospatial/executive functions, language, attention, naming, abstraction, orientation, and recall, and is widely used to screen for cognitive decline [18]. MoCA scores are adjusted for educational attainment. When a participant has less than 12 years of education, the MoCA score is increased by 1. The total MoCA score ranges from 0 to 30 points, with lower scores indicating poorer cognitive function.

### Statistical analysis

Statistical analysis was performed via SAS 9.4 software. Cumulative BP exposure was assessed via continuous and tripartite variables. The general characteristics of the participants were evaluated according to cumulative SBP tertiles and are expressed as count (percentage) for categorical variables, mean ± standard deviation for continuous variables if normally distributed, and median (interquartile range) if skewed. Generalized linear regression models were used to investigate the associations between cumulative BP exposure, CBF, brain volume, and cognitive function. All analyses in this study were based on the following 3 models. Model 1 was adjusted for sex and age at baseline. Model 2 was based on Model 1 and was further adjusted for baseline smoking status, drinking status, physical exercise, BMI, FBG level, LDL-C level, antihypertensive drug use, hypoglycemic drug use, lipid-lowering drug use, and total intracranial volume. Considering the possible changes in BMI, FBG levels, LDL-C levels and drug use during follow-up, the average BMI, FBG level, LDL-C level, antihypertensive drug use, hypoglycemic drug use, and lipid-lowering drug use during follow-up were used in Model 3 to replace the BMI, FBG level, LDL-C level, antihypertensive drug use, hypoglycemic drug use, and lipid-lowering drug use in Model 2. Because Model 3 is adjusted for all confounding variables, the result obtained from this model is reported as the main result. In all the statistical analyses, *P* < 0.05 was considered to indicate statistical significance. Multiple imputation by chained equations was used to compensate for missing values of covariates. The results are expressed with regression coefficients β and 95% confidence intervals.

To test the hypothesis that cumulative BP affects cognitive function through alterations in brain volume and CBF, mediation analyses were conducted via the SAS CAUSALMED procedure. Mediation analysis adopts asymptotic theory for maximum likelihood estimation and uses the bootstrap method based on resampling (Monte Carlo simulation times = 1000) to calculate confidence intervals (CIs) [19]. It quantifies the direct effect (the effect not affected by the mediator), indirect effect (the effect of the independent variable on the mediator multiplied by the effect of the mediator on the outcome), and total effect (the association between the independent variable and the outcome, which is equal to the sum of the direct effect and the indirect effect) [20]. The proportion mediated is calculated by dividing the natural indirect effect by the total effect. The analyses were adjusted for sex, age at baseline, smoking status, drinking status, physical exercise, mean BMI, mean FBG level, mean LDL-C level, antihypertensive drug use, hypoglycemic drug use, and lipid-lowering drug use during follow-up.

## Results

### Demographic characteristics

A total of 1012 participants were enrolled in this study, including 505 males and 507 females. The mean age at MRI examination was 57.76 ± 9.99 years. All participants underwent an average of 6 BP measurements, and the average time interval from the first physical examination to the head MRI examination was 14.69 ± 0.71 years. Table 1 shows the baseline characteristics of participants according to the cumulative SBP tertiles. The participants in the highest cumulative SBP tertile were more likely to be older men, were more likely to smoke, drink, exercise, take antihypertensive drugs, and use hypoglycemic drugs; had higher FBG levels, LDL-C levels, and BMIs (*P* < 0.05).

**Table 1:** Baseline characteristics of participants

Characteristics	Total	Tertile of cumulative systolic blood pressure			*P*
	(*n* = 1012)	Low (*n* = 337)	Intermediate (*n* = 338)	High (*n* = 337)	
Age, years	43.07 ± 9.91	38.70 ± 8.89	42.82 ± 9.02	47.69 ± 9.70	< 0.001^a^
Male, *n* (%)	505 (49.90)	97 (28.78)	183 (54.14)	225 (66.77)	< 0.001^b^
Smoking history, *n* (%)	228 (22.52)	33 (9.79)	85 (25.15)	110 (32.64)	< 0.001^b^
Drinking history, *n* (%)	364 (35.96)	73 (21.66)	144 (42.60)	147 (43.62)	< 0.001^b^
Physical exercise, *n* (%)	105 (10.38)	25 (7.42)	34 (10.06)	46 (13.65)	0.029^b^
Cumulative SBP, mm Hg×years	1858.83 ± 247.47	1599.74 ± 98.06	1839.85 ± 54.00	2136.96 ± 162.65	< 0.001^a^
Cumulative DBP, mm Hg × years	1174.17 ± 143.12	1035.26 ± 77.53	1178.76 ± 69.37	1308.48 ± 115.42	< 0.001^a^
FBG, mmol/L	5.14 ± 1.30	4.91 ± 0.61	5.14 ± 1.62	5.37 ± 1.39	< 0.001^a^
LDL-C, mmol/L	2.25 ± 0.80	2.09 ± 0.72	2.24 ± 0.83	2.42 ± 0.82	< 0.001^a^
BMI, kg/m^2^	24.47 ± 3.43	22.55 ± 3.02	24.92 ± 3.07	25.92 ± 3.30	< 0.001^a^
Antihypertensive treatment, *n* (%)	110 (10.87)	4 (1.19)	25 (7.40)	81 (24.04)	< 0.001^b^
Hypoglycemic treatment, *n* (%)	19 (1.88)	0	4 (1.18)	15 (4.45)	< 0.001^b^
Lipid-lowering treatment, *n* (%)	9 (0.89)	3 (0.89)	3 (0.89)	3 (0.89)	1.000 ^b^

Data are expressed as the mean ± standard deviation or number (percentage)

*SBP* systolic blood pressure, *DBP* diastolic blood pressure, *FBG* fasting blood glucose, *LDL-C* low-density lipoprotein cholesterol, *BMI* body mass index

^a^ One-way analysis of variance

^b^ chi-squared test

### Cumulative BP exposure and brain volume, CBF

**Fig. 2:**
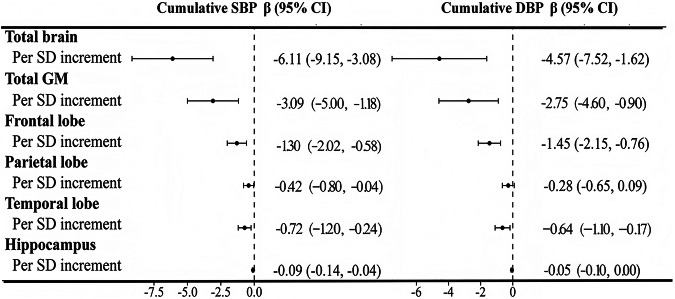
Associations between cumulative BP measurements and brain volume. All analyses were adjusted for sex, age at baseline, smoking status, drinking status, physical exercise, mean BMI, mean FBG level, mean LDL-C level, antihypertensive drug use, hypoglycemic drug use, and lipid-lowering drug use. BP blood pressure, SBP systolic blood pressure, DBP diastolic blood pressure, FBG fasting blood glucose, LDL-C low-density lipoprotein cholesterol, BMI body mass index, GM gray matter

**Fig. 3:**
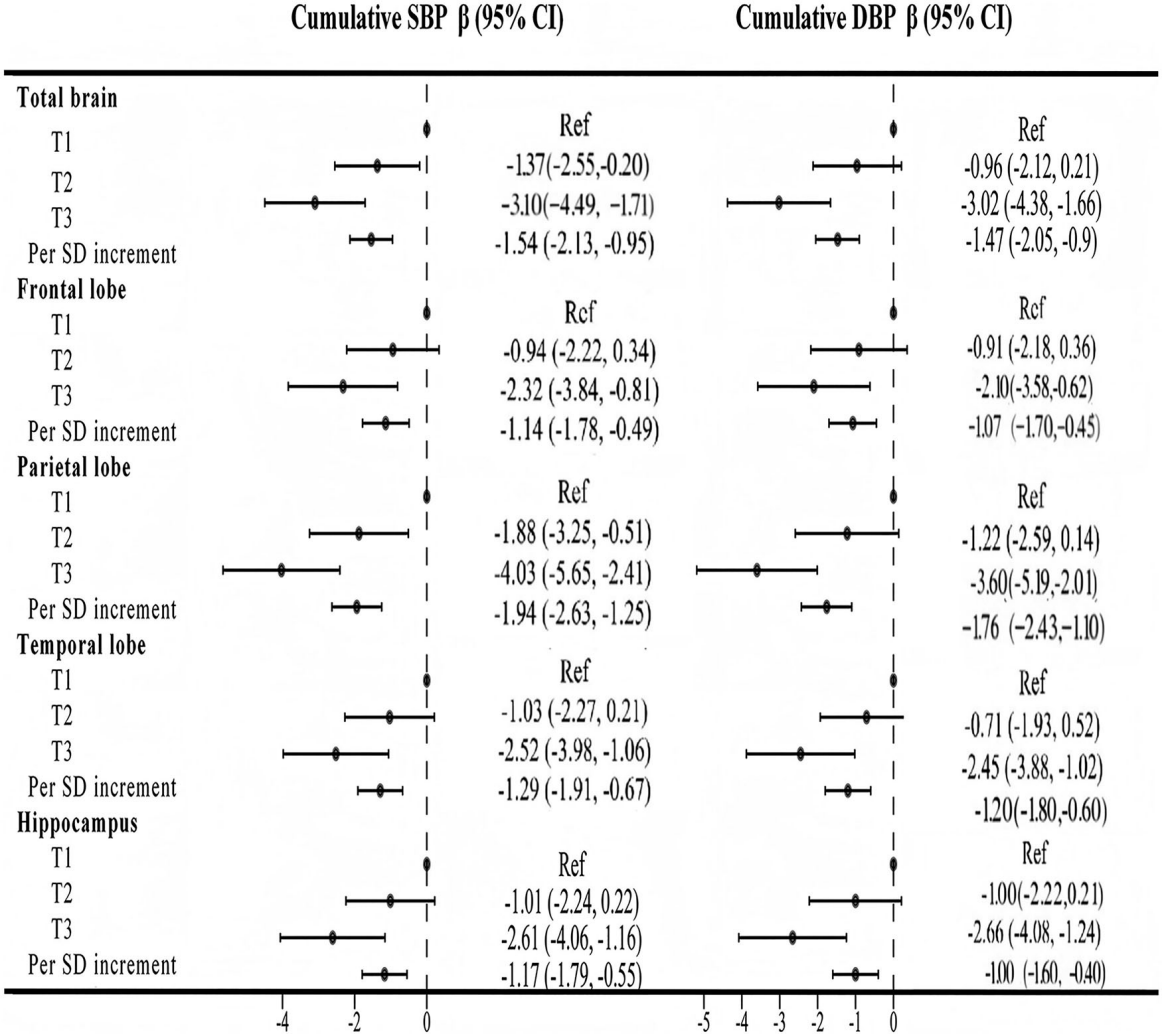
Associations between cumulative BP exposure and total and regional CBF. All analyses were adjusted for sex, age at baseline, smoking status, drinking status, physical exercise, mean BMI, mean FBG level, mean LDL-C level, antihypertensive drug use, hypoglycemic drug use, and lipid-lowering drug use. BP blood pressure, SBP systolic blood pressure, DBP diastolic blood pressure, FBG fasting blood glucose, LDL-C low-density lipoprotein cholesterol, BMI body mass index, GM gray matter, CBF cerebral blood flow

Figure 2 and Supplementary Table 1 show the associations between cumulative BP exposure and brain volume. Compared with the lowest tertiles, the highest cumulative SBP tertiles were associated with lower volume in total brain (β [95% CI]: −9.11 [−16.25, −1.97]), total GM (β [95% CI]: -5.53 [−10.02, −1.04]), frontal lobe (β [95% CI]: −2.46 [−4.15, −0.78]), temporal lobe (β [95% CI]: −1.37 [−2.51, −0.23]) and hippocampus (β [95% CI]: −0.15 [−0.26, −0.03]). The highest cumulative DBP tertiles were associated with the volume in frontal lobe (β [95% CI]: −2.33[−3.98, −0.68]) and temporal lobe (β [95% CI]: −1.15[-2.27, −0.04]) (Supplementary Table 1). When the cumulative BP was a continuous variable, for each 1-SD increase in cumulative SBP exposure, the total brain, total GM, frontal lobe, parietal lobe, temporal lobe and hippocampal volumes decreased by 6.11, 3.09, 1.30, 0.42, 0.72 and 0.09 cm^3^, respectively. With each 1-SD increase in cumulative DBP exposure, the total brain, total GM, frontal lobe, temporal lobe and hippocampal volumes decreased by 4.57, 2.75, 1.45, 0.64 and 0.05 cm^3^, respectively (Fig. 2). Figure 3 and Supplementary Table 2 show the associations between cumulative BP exposure and total and regional CBF. Compared with the lowest tertiles, the highest cumulative SBP tertiles were associated with lower CBF in the total brain (β [95% CI]: −3.10 [−4.49, −1.71]), frontal lobe (β [95% CI]: −2.32 [−3.84, -0.81]), parietal lobe (β [95% CI]: −4.03 [−5.65, −2.41]), temporal lobe (β [95% CI]: −2.52 [−3.98, −1.06]), and hippocampus (β [95% CI]: −2.61 [−4.06,−1.16]). Compared with the lowest tertiles, the highest cumulative DBP tertiles were associated with lower CBF in the total brain (β [95% CI]: −3.02 [−4.38, −1.66]), frontal lobe (β [95% CI]: −2.10 [−3.58, −0.62]), parietal lobe (β [95% CI]: −3.60 [−5.19, −2.01]), temporal lobe (β [95% CI]: −2.45 [-3.88, −1.02]), and hippocampus (β [95% CI]: −2.66[−4.08, −1.24]) (Supplementary Table 2). When the cumulative BP was a continuous variable, for each 1-SD increase in cumulative SBP exposure, the CBF in the total brain, frontal lobe, parietal lobe, temporal lobe, and hippocampus decreased by 1.54, 1.14, 1.94, 1.29, and 1.17 ml/100 g/min, respectively. With each 1-SD increase in cumulative DBP exposure, the CBF in the total brain, frontal lobe, parietal lobe, temporal lobe, and hippocampus decreased by 1.47, 1.07, 1.76, 1.20, and 1.00 ml/100 g/min, respectively (Fig. 3).

### Cumulative BP exposure and cognitive function

**Fig. 4:**
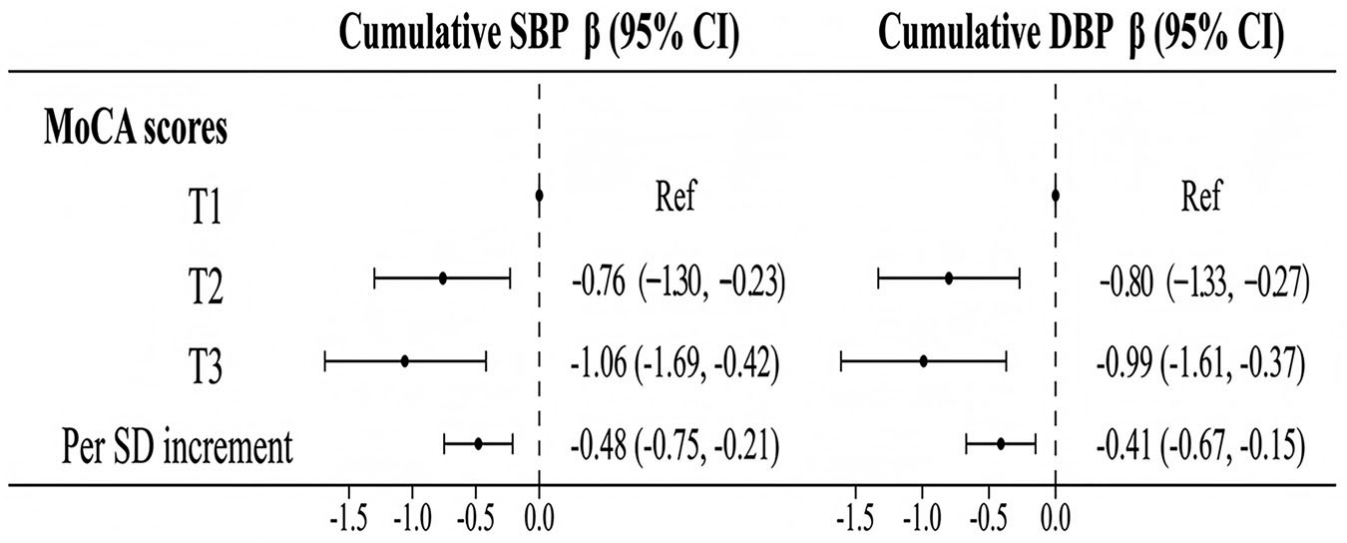
Associations between cumulative BP exposure and MoCA scores. All analyses were adjusted for sex, age at baseline, smoking status, drinking status, physical exercise, mean BMI, mean FBG level, mean LDL-C level, antihypertensive drug use, hypoglycemic drug use, and lipid-lowering drug use. BP blood pressure, SBP systolic blood pressure, DBP diastolic blood pressure, FBG fasting blood glucose, LDL-C low-density lipoprotein cholesterol, BMI body mass index, MoCA Montreal Cognitive Assessment

Figure 4 and Supplementary Table 3 show the associations between cumulative BP exposure and MoCA scores. Compared with the lowest tertiles, the highest cumulative SBP (β [95% CI]: −1.06 [−1.69, −0.42]) and cumulative DBP tertiles (β [95% CI]: −0.99 [−1.61, −0.37]) were associated with lower MoCA scores. When cumulative BP exposure was a continuous variable, for each 1-SD increase in cumulative SBP and DBP exposure, the MoCA scores decreased by 0.48 and 0.41, respectively.

### Brain volume, CBF and cognitive function

Supplementary Table 4 shows the associations among brain volume, CBF, and MoCA scores. For each 1-SD decrease in the total brain, total GM, frontal lobe, parietal lobe, temporal lobe, and hippocampal volumes, the MoCA scores decreased by 0.37, 0.48, 0.40, 0.39, 0.48 and 0.27, respectively. There were no significant associations between CBF and MoCA scores with respect to total or regional brain regions (*P* > 0.05).

### Mediation analysis

**Fig. 5:**
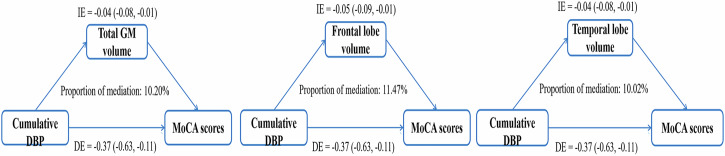
Mediation effect by brain volume in the association between cumulative blood pressure and cognitive function. DE direct effect, GM gray matter, IE indirect effect, SBP systolic blood pressure, DBP diastolic blood pressure, MoCA Montreal Cognitive Assessment

After adjusting for potential confounding factors, mediation analysis revealed that the associations between cumulative DBP exposure and MoCA scores were mediated by total GM volume (indirect effect: −0.04 [−0.08, −0.01]; direct effect: −0.37 [−0.63, −0.11]; proportion: 10.20%), frontal lobe volume (indirect effect: −0.05 [−0.09, −0.01]; direct effect: −0.37 [−0.63, −0.11]; proportion: 11.47%) and temporal lobe volume (indirect effect: −0.04 [−0.08, −0.01]; direct effect: −0.37 [−0.63, −0.11]; proportion: 10.02%) (Fig. 5). Altered brain volume did not mediate the association between cumulative SBP exposure and MoCA scores.

## Discussion

This study revealed that in the general population, cumulative SBP and DBP exposure were not only negatively associated with total and regional brain volume and CBF but also risk factors for cognitive decline. Decreased total brain, total GM, frontal lobe, parietal lobe, temporal lobe, and hippocampal volumes were significantly associated with lower MoCA scores. More importantly, decreased total GM, frontal lobe, and temporal lobe volumes mediated the association between cumulative DBP exposure and cognitive decline.

This study revealed that elevated cumulative BP exposure had disparate effects on total brain volume and regional brain volume. Both cumulative SBP and DBP exposure were negatively associated with total brain, total GM, frontal lobe, temporal lobe and hippocampal volumes. Additionally, cumulative SBP exposure was negatively associated with parietal lobe volume. These associations remained significant even after adjusting for potential confounding variables, including total intracranial volume and medication history. Furthermore, our findings indicated that cumulative SBP exposure exerted a more profound influence on the aforementioned brain regions than did cumulative DBP exposure. Hajjar et al. investigated the association between BP levels and brain volume in an elderly population and revealed a significant negative association between SBP and frontal‒parietal lobe volume [21]. A cohort study based on older adults (60–90 years old) without dementia demonstrated that in participants with untreated hypertension, elevated DBP was associated with a greater degree of hippocampal atrophy [22]. Additionally, Li et al. reported that both SBP and DBP were negatively associated with total and regional brain volume in the general adult population [13]. However, the above studies adopted single BP measurements, which cannot effectively reflect participants’ true BP levels within a certain period. This may be the cause of the resulting differences. In this study, we calculated the cumulative BP exposure over time using BP data across 14.7 years and investigated the associations between cumulative BP exposure and the volume of cognitively relevant brain regions in the general population. The hippocampus is the key brain region responsible for cognitive function. It is also the brain area that is most sensitive to fluctuations in BP [23]. We found that both higher cumulative SBP and DBP exposure are associated with lower hippocampal volume. Furthermore, our findings revealed a negative association between cumulative SBP exposure and parietal lobe volume, whereas no such association was observed with cumulative DBP exposure. Atrophy of the parietal lobe has been linked to both cognitive decline and the early stages of Alzheimer’s disease [24]. The results of our study indicate that the effect of cumulative SBP exposure on parietal lobe volume is specific. Furthermore, the present study investigated the associations between cumulative BP exposure and CBF. The findings revealed that both elevated cumulative SBP and DBP exposure resulted in a reduction in total and local CBF.

Although the underlying mechanisms of the associations between cumulative BP exposure and brain volume and perfusion remain unclear, the available literature reports that sustained BP elevation can lead to cerebral vascular remodeling by inducing damage to small artery endothelial cells [25] and the basement membrane [26] and a reduction in the number of vascular smooth muscle cells [27], leading to reduced CBF [28]. Consequently, sustained reductions in CBF result in endothelial dysfunction, oxidative stress, and aberrant protein expression, which in turn lead to further reductions in cerebral perfusion [23]. Second, elevated BP also disrupts the coordinated coupling among neurons, glial cells, and CBF in the cerebral microcirculation [29], affecting the transport of oxygen and glucose, which further leads to neuronal loss [30], resulting in a reduction in GM volume [31]. Furthermore, elevated BP can increase the number of peripheral blood inflammatory cytokines [32] through disruption of the blood‒brain barrier axis [33], resulting in damage to organs and the brain’s influence on the autonomic nervous system [34]. Additionally, the activation of glial cells associated with inflammation-induced nerve inflammation [35] can lead to additional structural and functional brain damage.

We found that cumulative SBP and DBP exposure were both inversely associated with cognitive decline after adjustment for conventional vascular risk factors. The Indo-US Cross National Dementia Epidemiology Study (INDO study) revealed that in participants older than 75 years, for every 10 mm Hg increase in DBP and SBP, the Mini-Mental State Examination (MMSE) score was reduced by more than 10% [36]. The Coronary Artery Risk Development in Young Adults (CARDIA) cohort study demonstrated that elevated cumulative SBP and DBP exposure in early adulthood was associated with diminished cognitive function in middle-aged individuals: for each 1-SD increase in cumulative SBP exposure, there was a statistically significant decrease in executive function, memory function, and global cognitive function, with effect sizes of 0.16, 0.12, and 0.12, respectively, for every 1-SD increase in cumulative DBP exposure, memory function decreased by 0.16 [10]. However, the above studies focused on specific ages in European and American individuals, some of whom were symptomatic patients. Our study, which was based on a general community population aged 29.7 to 83.9 years, confirmed the inverse association between cumulative BP exposure and cognitive decline. The underlying mechanism may be that long-term elevated cumulative BP exposure can lead to cerebral microcirculation disturbance and neuronal damage by reducing the vascular response, which in turn leads to cognitive decline [23]. Accordingly, the implementation of early intervention protocols pertaining to BP is instrumental in the attainment of cognitive function protection.

Importantly, decreased total GM, frontal lobe and temporal lobe volumes mediated the negative association between cumulative DBP exposure and cognitive function, and the mediating effects accounted for 10.199, 11.469 and 10.023% of the total effect, respectively. The GM is primarily composed of neurons, which are the fundamental units responsible for executing neural functions [37]. The temporal lobe serves as the network center for functions associated with memory, navigation, and time perception [38]. The frontal lobe is responsible for executive abilities, coordinating complex planning, organization, and multitasking activities [39]. A previous study demonstrated that for each 1-SD increase in DBP, the total GM volume, frontal lobe volume, and temporal lobe volume decreased by 4.04, 1.81, and 1.02, respectively [13]. In addition, DBP was negatively associated with cognitive functions, including memory and information processing speed [10, 40]. Both the frontal and temporal lobes are responsible for the processing of cognitive information [41, 42]. It can be reasonably deduced that prolonged exposure to elevated chronic SBP may result in cognitive decline through the impairment of neurons in the GM of these brain regions. Although no similar studies have been conducted, animal and human studies have confirmed that hypertension can not only lead to neuronal damage and apoptosis by inducing vascular dysfunction but also affect signal transmission between neurons, thereby affecting brain function [43, 44]. Increased DBP can also accelerate intracranial atherosclerosis and arteriolar sclerosis [45]. Therefore, long-term elevated cumulative DBP exposure can lead to further cognitive decline by causing damage to neurons in the supply region [46, 47].

Our study has several limitations. First, it should be noted that the present study was a cross-sectional study, which is unable to accurately reflect the causal associations among cumulative BP exposure, brain volume, CBF and cognitive function. Therefore, the mediation analysis in this study is exploratory and hypothesis-generating. Future longitudinal studies on the association between BP exposure, brain volume, CBF and cognitive function will be helpful in addressing this issue. Second, this study employed the MoCA to evaluate global cognitive function; however, this instrument may not be particularly sensitive to specific cognitive domains. Third, this study included an Asian population, and whether the findings of this study can be generalized to other populations remains unknown. Fourth, long-term cumulative BP exposure, which may ignore BP changes in the short term, was calculated in this study. Fifth, the types of antihypertensive drugs may have an impact on the association between cumulative BP, brain structure and cognitive function. However, detailed information on types of antihypertensive drugs was available for only a small subset of participants. In subsequent studies, we plan to collect medication information more comprehensively and further examine the potential effects of antihypertensive drug types on these associations.

In summary, we found that in the general population, cumulative BP exposure is negatively associated with total and regional brain volumes and CBF and is also a risk factor for cognitive decline. Decreased total and regional brain volumes are associated with cognitive decline. Mediation analysis revealed that decreased total brain, frontal lobe and temporal lobe volumes mediated the association between cumulative DBP exposure and cognitive decline. This study provides a theoretical basis for better understanding the harm of high cumulative BP exposure in clinical work and clarifies the importance of daily BP control. Our findings highlight that consistent control of BP can protect brain structure and function and prevent subsequent cognitive decline.

## Supplementary information

Supplementary information
