# Supplementary material for: Cumulative blood pressure exposure and cognition: the potential mediating role of brain volume
Source: Hypertens Res. 2026 Jan 14;49(4):1361–70. doi: 10.1038/s41440-025-02534-z (PMC13050639; doi:10.1038/s41440-025-02534-z)
Supplement: Supplementary file 1 — Supplementary information [file 41440_2025_2534_MOESM1_ESM.docx]

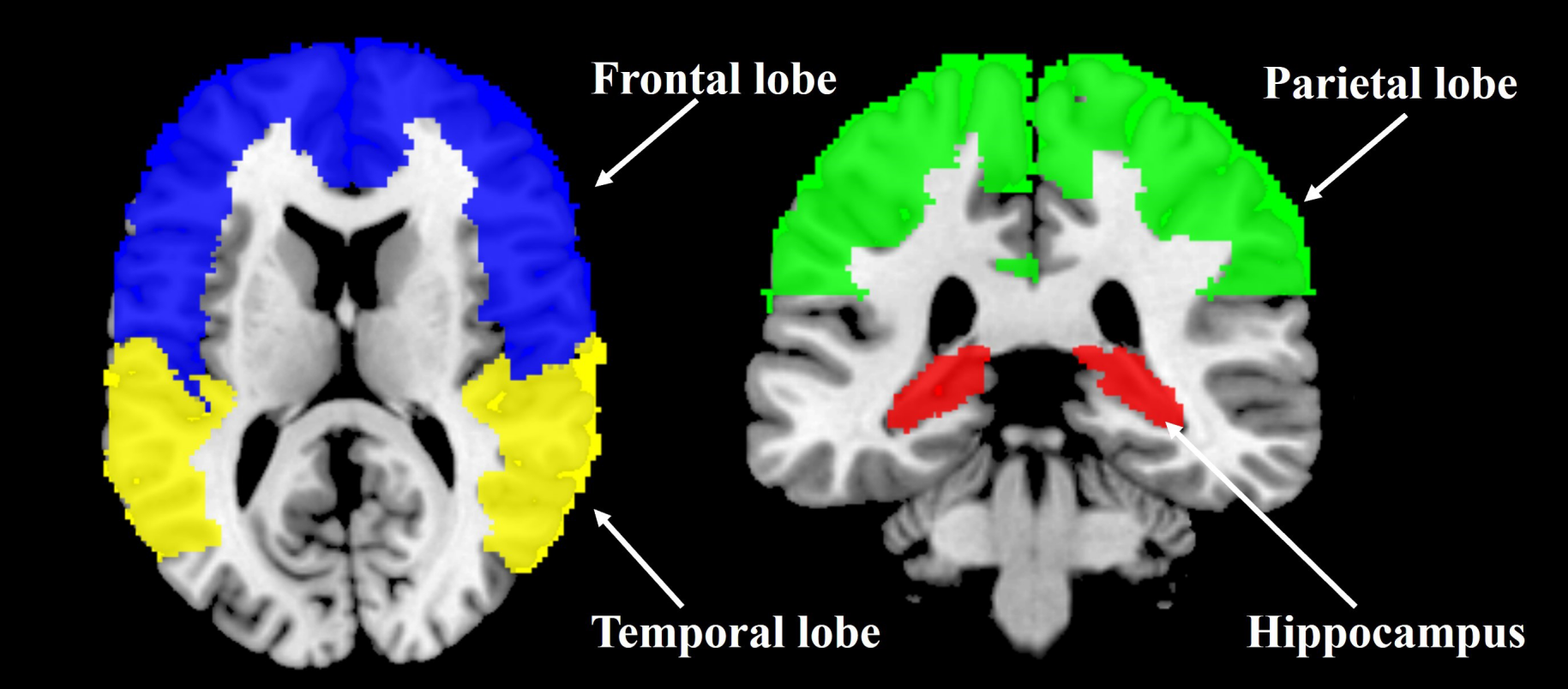


Supplementary Figure 1 Brain volume and perfusion of regions of interests were extracted

**Supplementary Table 1 Associations between cumulative BP exposure and brain volume.**

|  |  | Model 1 | | Model 2 | | Model 3 | |
| --- | --- | --- | --- | --- | --- | --- | --- |
|  | Tertile | β (95% CI) | *P* | β (95% CI) | *P* | β (95% CI) | *P* |
| **Total brain** |  |  |  |  |  |  |  |
| Cumulative SBP | T1 | Ref |  | Ref |  | Ref |  |
|  | T2 | -4.17 (-17.01, 8.67) | 0.524 | 1.40 (-4.49, 7.29) | 0.640 | 1.51 (-4.53, 7.55) | 0.623 |
|  | T3 | -8.50 (-22.37, 5.37) | 0.229 | -9.57 (-16.17, -2.96) | 0.005 | -9.11 (-16.25, -1.97) | 0.012 |
|  | Per SD increment | -5.50 (-11.22, 0.22) | 0.059 | -5.95 (-8.73, -3.17) | <0.001 | -6.11 (-9.15, -3.08) | <0.001 |
| Cumulative DBP | T1 | Ref |  | Ref |  | Ref |  |
|  | T2 | -11.25 (-24.05, 1.55) | 0.085 | -3.08 (-9.00, 2.85) | 0.309 | -1.57 (-7.58, 4.44) | 0.608 |
|  | T3 | -5.85 (-19.58, 7.87) | 0.403 | -6.92 (-13.47, -0.37) | 0.039 | -5.10 (-12.12, 1.92) | 0.154 |
|  | Per SD increment | -6.25 (-11.88, -0.61) | 0.030 | -5.10 (-7.81, -2.38) | <0.001 | -4.57 (-7.52, -1.62) | 0.003 |
| **Total GM** |  |  |  |  |  |  |  |
| Cumulative SBP | T1 | Ref |  | Ref |  | Ref |  |
|  | T2 | -4.28 (-10.67, 2.12) | 0.190 | -1.26 (-4.97, 2.45) | 0.506 | -1.01 (-4.80, 2.79) | 0.603 |
|  | T3 | -6.64 (-13.55, 0.26) | 0.059 | -6.40 (-10.57, -2.24) | 0.003 | -5.53 (-10.02, -1.04) | 0.016 |
|  | Per SD increment | -3.50 (-6.35, -0.65) | 0.016 | -3.34 (-5.09, -1.59) | <0.001 | -3.09 (-5.00, -1.18) | 0.002 |
| Cumulative DBP | T1 | Ref |  | Ref |  | Ref |  |
|  | T2 | -9.69 (-16.05, -3.32) | 0.003 | -5.43 (-9.15, -1.71) | 0.004 | -4.48 (-8.24, -0.72) | 0.020 |
|  | T3 | -5.12 (-11.94, 1.70) | 0.141 | -4.99 (-9.1, -0.88) | 0.018 | -3.65 (-8.05, 0.74) | 0.103 |
|  | Per SD increment | -4.00 (-6.80, -1.19) | 0.005 | -3.21 (-4.92, -1.51) | <0.001 | -2.75 (-4.60, -0.90) | 0.004 |
| **Frontal lobe** |  |  |  |  |  |  |  |
| Cumulative SBP | T1 | Ref |  | Ref |  | Ref |  |
|  | T2 | -1.22 (-3.32, 0.88) | 0.254 | -0.42 (-1.81, 0.96) | 0.549 | -0.14 (-1.56, 1.28) | 0.848 |
|  | T3 | -2.79 (-5.06, -0.52) | 0.016 | -3.02 (-4.58, -1.47) | 0.000 | -2.46 (-4.15, -0.78) | 0.004 |
|  | Per SD increment | -1.40 (-2.34, -0.47) | 0.003 | -1.51 (-2.17, -0.86) | <.0001 | -1.30 (-2.02, -0.58) | <0.001 |
| Cumulative DBP | T1 | Ref |  | Ref |  | Ref |  |
|  | T2 | -3.29 (-5.38, -1.21) | 0.002 | -2.16 (-3.55, -0.77) | 0.002 | -1.78 (-3.19, -0.36) | 0.014 |
|  | T3 | -2.73 (-4.97, -0.49) | 0.017 | -2.98 (-4.52, -1.44) | 0.000 | -2.33 (-3.98, -0.68) | 0.006 |
|  | Per SD increment | -1.76 (-2.68, -0.84) | <0.001 | -1.68 (-2.31, -1.04) | <.0001 | -1.45 (-2.15, -0.76) | <.0001 |
| **Parietal lobe** |  |  |  |  |  |  |  |
| Cumulative SBP | T1 | Ref |  | Ref |  | Ref |  |
|  | T2 | -0.50 (-1.5, 0.49) | 0.323 | -0.06 (-0.79, 0.67) | 0.874 | 0.05 (-0.71, 0.80) | 0.899 |
|  | T3 | -0.90 (-1.97, 0.18) | 0.102 | -0.78 (-1.60, 0.05) | 0.064 | -0.50 (-1.40, 0.39) | 0.268 |
|  | Per SD increment | -0.56 (-1.00, -0.11) | 0.014 | -0.50 (-0.84, -0.15) | 0.005 | -0.42 (-0.80, -0.04) | 0.031 |
| Cumulative DBP | T1 | Ref |  | Ref |  | Ref |  |
|  | T2 | -1.28 (-2.28, -0.29) | 0.011 | -0.70 (-1.43, 0.04) | 0.063 | -0.48 (-1.23, 0.27) | 0.207 |
|  | T3 | -0.69 (-1.76, 0.37) | 0.202 | -0.57 (-1.38, 0.24) | 0.168 | -0.32 (-1.20, 0.55) | 0.470 |
|  | Per SD increment | -0.54 (-0.98, -0.10) | 0.016 | -0.40 (-0.73, -0.06) | 0.022 | -0.28 (-0.65, 0.09) | 0.132 |
| **Temporal lobe** |  |  |  |  |  |  |  |
| Cumulative SBP | T1 | Ref |  | Ref |  | Ref |  |
|  | T2 | -1.01 (-2.39, 0.37) | 0.152 | -0.37 (-1.31, 0.57) | 0.439 | -0.23 (-1.19, 0.73) | 0.639 |
|  | T3 | -1.81 (-3.30, -0.32) | 0.018 | -1.66 (-2.70, -0.61) | 0.002 | -1.37 (-2.51, -0.23) | 0.018 |
|  | Per SD increment | -0.90 (-1.52, -0.29) | 0.004 | -0.82 (-1.26, -0.38) | <0.001 | -0.72 (-1.20, -0.24) | 0.004 |
| Cumulative DBP | T1 | Ref |  | Ref |  | Ref |  |
|  | T2 | -2.10 (-3.48, -0.73) | 0.003 | -1.21 (-2.15, -0.28) | 0.011 | -0.99 (-1.94, -0.03) | 0.043 |
|  | T3 | -1.61 (-3.09, -0.14) | 0.032 | -1.50 (-2.53, -0.46) | 0.005 | -1.15 (-2.27, -0.04) | 0.042 |
|  | Per SD increment | -0.98 (-1.58, -0.37) | 0.002 | -0.78 (-1.21, -0.35) | <0.001 | -0.64 (-1.10, -0.17) | 0.008 |
| **Hippocampus** |  |  |  |  |  |  |  |
| Cumulative SBP | T1 | Ref |  | Ref |  | Ref |  |
|  | T2 | 0.02 (-0.08, 0.12) | 0.697 | 0.02 (-0.07, 0.12) | 0.620 | -0.01 (-0.10, 0.09) | 0.911 |
|  | T3 | -0.08 (-0.19, 0.04) | 0.189 | -0.10 (-0.21, 0.00) | 0.059 | -0.15 (-0.26, -0.03) | 0.012 |
|  | Per SD increment | -0.05 (-0.10, 0.00) | 0.033 | -0.06 (-0.11, -0.02) | 0.005 | -0.09 (-0.14, -0.04) | <0.001 |
| Cumulative DBP | T1 | Ref |  | Ref |  | Ref |  |
|  | T2 | -0.01 (-0.12, 0.09) | 0.804 | 0.00 (-0.09, 0.10) | 0.967 | -0.01 (-0.11, 0.08) | 0.798 |
|  | T3 | 0.00 (-0.12, 0.11) | 0.942 | -0.04 (-0.14, 0.07) | 0.483 | -0.06 (-0.17, 0.05) | 0.312 |
|  | Per SD increment | -0.03 (-0.08, 0.02) | 0.190 | -0.04 (-0.08, 0.00) | 0.076 | -0.05 (-0.10, 0.00) | 0.033 |

Associations between cumulative BP measurements and brain volume. All analyses were adjusted for sex, age at baseline, smoking status, drinking status, physical exercise, mean BMI, mean FBG level, mean LDL-C level, antihypertensive drug use, hypoglycemic drug use, and lipid-lowering drug use. Abbreviation: BP, blood pressure; SBP, systolic blood pressure; DBP, diastolic blood pressure; FBG, fasting blood glucose; LDL-C, low-density lipoprotein cholesterol; BMI, body mass index; GM, gray matter.

**Supplementary Table 2 Associations between cumulative BP exposure and total and regional CBF.**

|  |  | Model 1 | | Model 2 | | Model 3 | |
| --- | --- | --- | --- | --- | --- | --- | --- |
|  | Tertile | β (95% CI) | *P* | β (95% CI) | *P* | β (95% CI) | *P* |
| **Total brain** |  |  |  |  |  |  |  |
| Cumulative SBP | T1 | Ref |  | Ref |  | Ref |  |
|  | T2 | -1.52 (-2.63, -0.42) | 0.007 | -1.47 (-2.62, -0.33) | 0.012 | -1.37 (-2.55, -0.20) | 0.022 |
|  | T3 | -3.23 (-4.42, -2.04) | <0.001 | -3.10 (-4.39, -1.82) | <0.001 | -3.10 (-4.49, -1.71) | <0.001 |
|  | Per SD increment | -1.48 (-1.97, -1.00) | <0.001 | -1.47 (-2.01, -0.93) | <0.001 | -1.54 (-2.13, -0.95) | <0.001 |
| Cumulative DBP | T1 | Ref |  | Ref |  | Ref |  |
|  | T2 | -1.08 (-2.17, 0.02) | 0.055 | -1.04 (-2.19, 0.10) | 0.074 | -0.96 (-2.12, 0.21) | 0.108 |
|  | T3 | -3.24 (-4.42, -2.06) | <0.001 | -3.05 (-4.32, -1.79) | <0.001 | -3.02 (-4.38, -1.66) | <0.001 |
|  | Per SD increment | -1.48 (-1.96, -1.00) | <0.001 | -1.45 (-1.98, -0.93) | <0.001 | -1.47 (-2.05, -0.90) | <0.001 |
| **Frontal lobe** |  |  |  |  |  |  |  |
| Cumulative SBP | T1 | Ref |  | Ref |  | Ref |  |
|  | T2 | -1.31 (-2.50, -0.11) | 0.033 | -1.16 (-2.40, 0.09) | 0.069 | -0.94 (-2.22, 0.34) | 0.150 |
|  | T3 | -2.82 (-4.11, -1.53) | <0.001 | -2.57 (-3.97, -1.18) | <0.001 | -2.32 (-3.84, -0.81) | 0.003 |
|  | Per SD increment | -1.29 (-1.82, -0.75) | <0.001 | -1.21 (-1.79, -0.62) | <0.001 | -1.14 (-1.78, -0.49) | 0.001 |
| Cumulative DBP | T1 | Ref |  | Ref |  | Ref |  |
|  | T2 | -1.21 (-2.41, -0.02) | 0.046 | -1.05 (-2.30, 0.20) | 0.099 | -0.91 (-2.18, 0.36) | 0.158 |
|  | T3 | -2.73 (-4.01, -1.44) | <0.001 | -2.37 (-3.75, -0.99) | 0.001 | -2.10 (-3.58, -0.62) | 0.005 |
|  | Per SD increment | -1.27 (-1.80, -0.75) | <0.001 | -1.16 (-1.73, -0.59) | <0.001 | -1.07 (-1.70, -0.45) | 0.001 |
| **Parietal lobe** |  |  |  |  |  |  |  |
| Cumulative SBP | T1 | Ref |  | Ref |  | Ref |  |
|  | T2 | -2.06 (-3.35, -0.78) | 0.002 | -2.03 (-3.37, -0.69) | 0.003 | -1.88 (-3.25, -0.51) | 0.007 |
|  | T3 | -4.17 (-5.56, -2.79) | <0.001 | -4.11 (-5.60, -2.61) | <0.001 | -4.03 (-5.65, -2.41) | <0.001 |
|  | Per SD increment | -1.89 (-2.46, -1.32) | <0.001 | -1.92 (-2.54, -1.29) | <0.001 | -1.94 (-2.63, -1.25) | <0.001 |
| Cumulative DBP | T1 | Ref |  | Ref |  | Ref |  |
|  | T2 | -1.40 (-2.69, -0.12) | 0.032 | -1.40 (-2.74, -0.06) | 0.041 | -1.22 (-2.59, 0.14) | 0.078 |
|  | T3 | -3.93 (-5.30, -2.55) | <0.001 | -3.78 (-5.26, -2.30) | <0.001 | -3.60 (-5.19, -2.01) | <0.001 |
|  | Per SD increment | -1.80 (-2.37, -1.24) | <0.001 | -1.79 (-2.41, -1.18) | <0.001 | -1.76 (-2.43, -1.10) | <0.001 |
| **Temporal lobe** |  |  |  |  |  |  |  |
| Cumulative SBP | T1 | Ref |  | Ref |  | Ref |  |
|  | T2 | -1.28 (-2.44, -0.13) | 0.030 | -1.16 (-2.37, 0.04) | 0.059 | -1.03 (-2.27, 0.21) | 0.102 |
|  | T3 | -2.82 (-4.07, -1.57) | <0.001 | -2.59 (-3.94, -1.24) | <0.001 | -2.52 (-3.98, -1.06) | 0.001 |
|  | Per SD increment | -1.32 (-1.83, -0.80) | <0.001 | -1.25 (-1.82, -0.68) | <0.001 | -1.29 (-1.91, -0.67) | <0.001 |
| Cumulative DBP | T1 | Ref |  | Ref |  | Ref |  |
|  | T2 | -0.92 (-2.08, 0.23) | 0.118 | -0.80 (-2.01, 0.40) | 0.192 | -0.71 (-1.93, 0.52) | 0.258 |
|  | T3 | -2.84 (-4.08, -1.60) | <0.001 | -2.52 (-3.85, -1.19) | <0.001 | -2.45 (-3.88, -1.02) | 0.001 |
|  | Per SD increment | -1.29 (-1.8, -0.78) | <0.001 | -1.20 (-1.75, -0.64) | <0.001 | -1.20 (-1.80, -0.60) | <0.001 |
| **Hippocampus** |  |  |  |  |  |  |  |
| Cumulative SBP | T1 | Ref |  | Ref |  | Ref |  |
|  | T2 | -1.02 (-2.17, 0.13) | 0.083 | -0.95 (-2.15, 0.25) | 0.120 | -1.01 (-2.24, 0.22) | 0.107 |
|  | T3 | -2.53 (-3.77, -1.29) | <0.001 | -2.38 (-3.72, -1.04) | 0.001 | -2.61 (-4.06, -1.16) | <0.001 |
|  | Per SD increment | -1.08 (-1.59, -0.56) | <0.001 | -1.02 (-1.58, -0.45) | <0.001 | -1.17 (-1.79, -0.55) | <0.001 |
| Cumulative DBP | T1 | Ref |  | Ref |  | Ref |  |
|  | T2 | -1.07 (-2.22, 0.08) | 0.068 | -1.02 (-2.22, 0.18) | 0.095 | -1.00 (-2.22, 0.21) | 0.106 |
|  | T3 | -2.58 (-3.81, -1.35) | <0.001 | -2.41 (-3.74, -1.09) | <0.001 | -2.66 (-4.08, -1.24) | <0.001 |
|  | Per SD increment | -0.98 (-1.49, -0.47) | <0.001 | -0.90 (-1.45, -0.35) | 0.001 | -1.00 (-1.60, -0.40) | 0.001 |

Associations between cumulative BP exposure and total and regional CBF. All analyses were adjusted for sex, age at baseline, smoking status, drinking status, physical exercise, mean BMI, mean FBG level, mean LDL-C level, antihypertensive drug use, hypoglycemic drug use, and lipid-lowering drug use. Abbreviation: BP, blood pressure; SBP, systolic blood pressure; DBP, diastolic blood pressure; FBG, fasting blood glucose; LDL-C, low-density lipoprotein cholesterol; BMI, body mass index; GM, gray matter; CBF, cerebral blood flow.

**Supplementary Table 3 Associations between cumulative BP exposure and Moca scores.**

|  |  | Model 1 | | Model 2 | | Model 3 | |
| --- | --- | --- | --- | --- | --- | --- | --- |
|  | Tertile | OR (95% CI) | *P* | OR (95% CI) | *P* | OR (95% CI) | *P* |
| **MoCA score** |  |  |  |  |  |  |  |
| Cumulative SBP | T1 | Ref |  | Ref |  | Ref |  |
|  | T2 | -0.79 (-1.30, -0.29) | 0.002 | -0.78 (-1.30, -0.26) | 0.003 | -0.76 (-1.30, -0.23) | 0.005 |
|  | T3 | -1.13 (-1.67, -0.58) | <0.001 | -1.16 (-1.74, -0.58) | <0.001 | -1.06 (-1.69, -0.42) | 0.001 |
|  | Per SD increment | -0.48 (-0.71, -0.26) | <0.001 | -0.51 (-0.76, -0.27) | <0.001 | -0.48 (-0.75, -0.21) | 0.001 |
| Cumulative DBP | T1 | Ref |  | Ref |  | Ref |  |
|  | T2 | -0.84 (-1.34, -0.34) | 0.001 | -0.76 (-1.28, -0.24) | 0.004 | -0.80 (-1.33, -0.27) | 0.003 |
|  | T3 | -1.09 (-1.63, -0.55) | <0.001 | -1.06 (-1.64, -0.48) | <0.001 | -0.99 (-1.61, -0.37) | 0.002 |
|  | Per SD increment | -0.43 (-0.65, -0.21) | <0.001 | -0.43 (-0.67, -0.19) | <0.001 | -0.41 (-0.67, -0.15) | 0.002 |

Associations between cumulative BP exposure and MoCA scores. All analyses were adjusted for sex, age at baseline, smoking status, drinking status, physical exercise, mean BMI, mean FBG level, mean LDL-C level, antihypertensive drug use, hypoglycemic drug use, and lipid-lowering drug use. Abbreviation: BP, blood pressure; SBP, systolic blood pressure; DBP, diastolic blood pressure; FBG, fasting blood glucose; LDL-C, low-density lipoprotein cholesterol; BMI, body mass index; MoCA, Montreal Cognitive Assessment.

**Supplementary Table 4 Associations between brain volume, CBF, and Moca scores.**

|  | Model 1 | | Model 2 | | Model 3 | |
| --- | --- | --- | --- | --- | --- | --- |
|  | β (95% CI) | *P* | β (95% CI) | *P* | β (95% CI) | *P* |
| **MoCA score** |  |  |  |  |  |  |
| **Brain volume** |  |  |  |  |  |  |
| Total brain | 0.33 (0.08, 0.58) | 0.010 | 0.37 (0.12, 0.62) | 0.004 | 0.37 (0.11, 0.62) | 0.005 |
| Total GM | 0.45 (0.20, 0.70) | <0.001 | 0.48 (0.23, 0.73) | <0.001 | 0.48 (0.23, 0.73) | <0.001 |
| Frontal lobe | 0.39 (0.15, 0.63) | 0.001 | 0.41 (0.17, 0.65) | 0.001 | 0.40 (0.16, 0.64) | 0.001 |
| Parietal lobe | 0.37 (0.14, 0.61) | 0.002 | 0.40 (0.16, 0.63) | 0.001 | 0.39 (0.15, 0.63) | 0.001 |
| Temporal lobe | 0.47 (0.21, 0.72) | <0.001 | 0.49 (0.23, 0.75) | <0.001 | 0.48 (0.22, 0.74) | <0.001 |
| Hippocampus | 0.23 (-0.01, 0.47) | 0.064 | 0.26 (0.02, 0.51) | 0.033 | 0.27 (0.03, 0.52) | 0.028 |
| **Brain perfusion** |  |  |  |  |  |  |
| Total brain | 0.03 (-0.17, 0.24) | 0.735 | 0.03 (-0.17, 0.23) | 0.752 | 0.03 (-0.17, 0.23) | 0.791 |
| Frontal lobe | 0.07 (-0.13, 0.27) | 0.516 | 0.06 (-0.14, 0.27) | 0.528 | 0.05 (-0.15, 0.25) | 0.616 |
| Parietal lobe | 0.07 (-0.13, 0.27) | 0.516 | 0.06 (-0.14, 0.27) | 0.528 | 0.05 (-0.15, 0.25) | 0.616 |
| Temporal lobe | 0.03 (-0.17, 0.23) | 0.789 | 0.02 (-0.18, 0.22) | 0.842 | 0.02 (-0.18, 0.22) | 0.854 |
| Hippocampus | 0.13 (-0.07, 0.33) | 0.215 | 0.12 (-0.08, 0.32) | 0.244 | 0.13 (-0.07, 0.33) | 0.207 |

Associations between brain volume, CBF, and MoCA scores. All analyses were adjusted for sex, age at baseline, smoking status, drinking status, physical exercise, mean BMI, mean FBG level, mean LDL-C level, antihypertensive drug use, hypoglycemic drug use, and lipid-lowering drug use. Abbreviation: BP, blood pressure; SBP, systolic blood pressure; DBP, diastolic blood pressure; FBG, fasting blood glucose; LDL-C, low-density lipoprotein cholesterol; BMI, body mass index; MoCA, Montreal Cognitive Assessment; GM, gray matter.
